# Significance of hematologic abnormalities in COVID-19 severity among infected patients in Lagos, Nigeria

**DOI:** 10.1186/s42269-022-00959-x

**Published:** 2022-12-02

**Authors:** Olufemi S. Amoo, Ngozi Onyia, Tochukwu I. Onuigbo, Stephanie U. Vitalis, Olabisi F. Davies-Bolorunduro, Joy I. Oraegbu, Esther T. Adeniji, Josephine C. Obi, Olusola N. Abodunrin, Amaka S. Ikemefuna, Richard A. Adegbola, Rosemary A. Audu, Babatunde L. Salako

**Affiliations:** 1 Nigerian Institute of Medical Research, Lagos, Nigeria; 2 Paelon Memorial Hospital, Lagos, Nigeria

**Keywords:** Severity, COVID-19, Neutrophilia, Lymphocytopenia, Anemia, Prognosis

## Abstract

**Background:**

There have been suggestions that hematologic abnormalities in COVID-19 are linked with the progression and severity of diseases and mortality. Lymphopenia, sepsis, and thrombocytopenia were highly reported in patients with COVID-19. This study investigated the significance of hematologic abnormalities in patients with COVID-19 in Lagos, Nigeria, and its potential as a diagnostic tool for COVID-19 severity.

**Results:**

This was a retrospective observational study with a total of 340 patients with COVID-19 (236 patients included in the analysis). These patients were categorized into two groups, comprising 71 patients with severe COVID-19 (SCP) and 165 patients with non-severe COVID-19 (NSCP). The majority were males in both categories (SCP 74.6% and NSCP 63.6%). The mean ± SD ages for SCP and NSCP were 52.28 ± 16.87 and 42.44 ± 17.18 years, respectively. The SCP (52.1%) and NSCP (20.0%) had underlying health conditions. The SCP exhibited significantly higher neutrophil counts (*P* < 0.05) and significantly lower mean hemoglobin, red blood cell (RBC), packed cell volume (PCV), and lymphocyte values (*P* < 0.05). Anemia and lymphocytopenia were more prominent in the SCP group than in the NSCP group (*P* < 0.05). Hemoglobin, RBC, PCV, and lymphocytes were inversely correlated with age-group in the SCP, while only lymphocytes and platelets were inversely correlated with age-group in the NSCP. The highest area under the ROC curve (AUC) for neutrophils was 0.739 with a sensitivity of 62.0% and specificity of 80.0%, while white blood cells had an AUC of 0.722 with a sensitivity of 73.2% and specificity of 61.2%. The AUC for neutrophil–lymphocyte ratio (NLR) was 0.766 with a sensitivity of 63.3% and specificity of 83.5%, while that for the platelet–lymphocyte ratio (PLR) was 0.695 with a sensitivity and specificity of 61.7% and 77.8%.

**Conclusions:**

COVID-19 affected the levels of hemoglobin, RBC, PCV, and lymphocytes in the blood, and the differences were significant between the SCP and NSCP. The significant changes in neutrophil and lymphocyte counts may be useful in the prognosis and management of COVID-19 severity in hospital settings. Furthermore, NLR and PLR may be used in the prognosis and management of severe COVID-19 infection, as well as provide an objective basis for early identification and management in low-resource settings.

## Background

SARS-COV 2, as the etiological agent of COVID-19, causes severe acute respiratory syndrome and is associated with multiple organ failure and death; it has rapidly evolved into a global pandemic affecting millions of people (Terpos et al. 2020). The disease has affected 222 countries/regions worldwide to date, and according to the World Health Organization (WHO), there have been approximately 540,562,504 confirmed cases and 6,331,506 deaths globally as of June 13, 2022 (WHO 2022). Although it was initially identified as a respiratory tract infection, emerging studies indicated that COVID-19 results in an illness associated with a wide range of clinical features, ranging from mild to moderate upper respiratory tract infections to severe systemic diseases (Mehta et al. 2020; Tang et al. 2020).

The initial symptoms of the disease include cough, colon and dyspnea, fatigue, fever, muscle pain, myalgia, and shortness of breath. They may progress to acute respiratory distress syndrome (ARDS), multi-organ dysfunction, shock, and metabolic acidosis if the condition worsens (Salehi-Abari et al. 2020). COVID-19 severity was divided into four levels (critical, severe, moderate, and mild) based on clinical manifestations, according to the National Health Commission of China's published protocol (version7) for diagnosis and treatment of COVID-19 (NHC and NATCM 2020).

Studies revealed that patients with clinical symptoms frequently progressed to pneumonia, with radiological proof of parenchymal disease (Shang et al. 2020; Yu et al. 2020). Another study suggested that individuals presenting with severe symptoms were likely to develop cytokine storm effect, as patients admitted to intensive care units showed elevated plasma levels of proinflammatory cytokines including interleukins and tumor necrosis factor (Huang et al. 2020). Hematologic abnormalities in COVID-19 have been linked with the progression and severity of disease and mortality (Taj et al. 2021). In addition, abnormal coagulation profile, lymphopenia, sepsis, and thrombocytopenia were highly reported in patients with COVID-19 infection (Lillicrap 2020). Lymphopenia was reported in about 35–85% of patients and was the most prevalent blood count abnormality (Yang et al. 2020a, b).

Platelet counts were independently associated with disease severity and risk of mortality in the intensive care unit (ICU) (Lippi et al. 2020). COVID-19 had been linked to coagulopathies such as arterial thrombotic complications, disseminated intravascular coagulation, local micro-thrombi, sepsis-induced coagulopathy (SIC), thrombo-inflammation, and venous thromboembolism (VTE) (Amgalan and Othman 2020). Hematologic biomarkers such as neutrophil and lymphocyte counts can play an important role in predicting disease severity early and provide a better guide for prompt patient management, thereby reducing disease morbidity and mortality. It is important to closely monitor the hematologic manifestations of this particular virus because the pandemic is still evolving.

In this study, we investigated the significance of hematologic abnormalities as diagnostic modalities in patients with COVID-19 infection in Lagos, Nigeria.

## Methods

### Study design

This was a retrospective observational study with a population of 340 individuals representing those seeking treatment for COVID-19 at a hospital-based isolation center in Lagos, Nigeria, from June to December 2020. Only 236 patients with complete hematologic and demographic data were included in this study, while 104 patients with incomplete demographic and hematologic data were excluded. COVID-19 test was performed on nasopharyngeal and throat swab samples using Roche Cobas® SARS-COV-2 test kit (Rotkreuz Switzerland) on Cobas 6800 system. Venous blood samples were collected from patients and analyzed for malaria parasite using “First Response® Malaria Antigen P. falciparum (HRP2) Card Test” (Premier Medical Corporation Private Limited, Valsad, Gujarat India), as well as hematologic parameters using automated hematology analyzer. Patient charts were reviewed, and the data collected for this study included the following: demographic information and hematologic parameters such as hemoglobin (Hb), red blood cell count (RBC), white blood cell count (WBC) or leukocytes, platelets (PLT), neutrophils (Neu), and lymphocytes (Lym) of patients who tested positive for COVID-19.

Based on the severity of patients with COVID-19, we classified 165 as “Patients with non-severe COVID-19” (NSCP) and 71 as “Patients with severe COVID-19” (SCP) according to WHO criteria (WHO 2020). Mild and moderate symptoms were classified as “non-severe,” while severe and critical were classified as “severe.”

*Inclusion criteria* (a) Patients diagnosed with COVID-19 according to WHO guidelines (Table 1) with positive SARS-COV-2 RT PCR test. (b) Patients whose hematologic data were available.

**Table 1:** WHO guidelines for COVID-19 severity classification. *Source*: WHO (2020)

Category	Symptoms
Mild	Fever, cough, fatigue, such as sore throat, nasal congestion, headache
Moderate	Adolescent or adult with clinical signs of pneumonia (fever, cough, dyspnea, fast breathing) but no signs of severe pneumonia, including SpO2 ≥ 90% on room air, loss of smell, loss of taste, diarrhea, nausea and vomiting, myalgia
Severe	Adolescent or adult with clinical signs of pneumonia (fever, cough, dyspnea, fast breathing) plus one of the following: respiratory rate > 30 breaths/min; severe respiratory distress; or SpO2 < 90% on room air. Loss of smell, loss of taste, diarrhea, nausea and vomiting, myalgia
Critical	Chest imaging: (radiograph, CT scan, or lung ultrasound) bilateral opacities, not fully explained by volume overload, lobar or lung collapse, or nodules Mild ARDS (invasively ventilated): 4 ≤ OI < 8 or 5 ≤ OSI < 7.5 Moderate ARDS (invasively ventilated): 8 ≤ OI < 16 or 7.5 ≤ OSI < 12.3 Severe ARDS (invasively ventilated): OI ≥ 16 or OSI ≥ 12.3

*ARDS* Acute respiratory disease syndrome, *CT* Computerized tomography, *OSI* Oxygen Saturation Index, *OI* Oxygenation Index

*Exclusion criteria* Patients with COVID-19 without documented hematology results.

*Data collection* A template was developed to collect specific information and data on the socio-demographic characteristics and hematologic parameters of patients with COVID-19 at Paelon Memorial Hospital.

*Data management and statistical analysis* Data editing, sorting, coding, classification, and tabulation were performed using Microsoft Excel. The hematologic parameters were presented as the mean ± standard deviation (mean ± SD). An independent t test was used to assess differences in mean values of severity levels of COVID-19 for hematologic parameters. The diagnostic performance of the studied parameters was evaluated using receiver operating characteristic (ROC) analysis; the odds ratio was also obtained. IBM SPSS software (version 23.0) was used for descriptive, independent sample t test for continuous variables and receiver operating characteristic analysis. Pearson chi-squared and the Fisher’s exact tests were used for categorical variables and logistic regression in this study. Bonferroni multiple-comparison correction for accurate p values was also performed. We considered significant statistical variations or associations at a *p* value of < 0.05.

## Results

### Social and demographic characteristics of study participants

The descriptive statistics for all the patients with COVID-19 were summarized and are presented in Table 2. Among the 236 participants, 158 (66.9%) were males and 78 (33.1%) were females. The mean ± SD age of the SCP was 52.3 ± 16.9 years and 42.4 ± 17.2 years for the NSCP. Most of the participants were between 24 and 65 years of age.

**Table 2:** Demographic characteristics and underlying conditions of patients with COVID-19

Parameters	NSCP (n = 165)			SCP (n = 71)			χ^2^	*P* value (< 0.05)
	n	%	Mean ± SD	n	%	Mean ± SD		
*Sex*								
Male	105	63.6		53	74.6			
Female	60	36.4		18	25.4			
*Nationality*								
Nigerian	110	66.7		42	59.2			
Non-Nigerian	55	33.3		29	40.8			
*Age in years*			42.4 ± 17.2			52.3 ± 16.9		
3–23	17	10.3		1	1.4			
24–44	82	49.7		25	35.2			
45–65	53	32.1		27	38.0			
66–86	13	7.9		18	25.4			
*Presence of comorbid disease*								
Yes	33	20.0		37	52.1		23.0208	0.000
No	132	80.0		34	47.9			
*Number of comorbid diseases*								
None	132	80.0		34	47.9			
One	22	13.3		20	28.2			
Two or more	11	6.7		17	23.9			
*Common comorbidities*								
Diabetes	10	6.0		11	15.5			
Hypertension	21	12.7		27	38.0			
Asthma	7	4.2		2	2.8			
*Mortality*								
Yes	0	0		6	8.5		11.100	0.000
No	165	100		65	91.5			
*Comorbidities associated with mortality*								
Diabetes and Hypertension	0	0		3	50			
Hypertension and ischemic heart disease	0	0		1	66.7			
Asthma	0	0		1	16.7			
Morbid obesity	0	0		1	16.7			
*Malaria parasite*	n = 73			n = 50				
Positive	1	1.4		3	6.0		0.8181	0.183
Negative	72	98.6		47	94.0			

*SCP *patients with severe COVID-19, *NSCP* patients with non-severe COVID-19

The *P* value is provided for comparison between the NSCP and SCP groups

Further analysis also revealed that 46 (65%) of SCP above 60 years of age were males, while 25 (35%) were females. Based on nationality, 152 (64.4%) were Nigerians, while 84 (35.6%) were non-Nigerians. Among the SCP, 37 (52.1%) had underlying conditions, of which 20 (28.2%) had one underlying condition and 17 (23.9%) had two or more underlying conditions. Common comorbidities were diabetes mellitus, hypertension, and asthma. It was observed that 22 (13.3%) of the NSCP had a history of one comorbid disease, with 11 (6.7%) having a history of two or more diseases.

While mortality and comorbidity were significantly different between the SCP and NSCP, the presence of malaria parasite was not. The SCP had a mortality of 6 (8.5%), but there were no mortalities in the NSCP. Major comorbidities associated with mortality were diabetes, hypertension, asthma, ischemic heart disease, and morbid obesity. A total of 123 patients were tested for malaria parasite with only 4 (3.25%) testing positive for the parasite. The members of the NSCP “group” were not hospitalized; however, the SCP had an average of 13-day length of stay in the facility.

### Hematologic abnormalities in patients with COVID-19

The hematologic alterations of the groups are presented in Table 3. First, a comparison was made between the hematologic variations of SCP and NSCP groups. The SCP had lower RBC and Hb levels than NSCP. The NSCP showed significantly lower WBC and neutrophil counts than SCP. It was also observed that PCV and lymphocyte counts were higher in NSCP than in SCP. The mean difference of Hb levels, RBC, PCV, WBC, neutrophil, lymphocyte, MID, and platelet counts was statistically significant at p value < 0.05. Bonferroni multiple-comparison correction test for Table 3 showed a p value of 0.005; this implied that all hematologic parameters with significant p value at < 0.05 were truly significant except for MID% and platelets (PLT).

**Table 3:** Hematologic parameters of the study groups at different severity levels of COVID-19

Parameters	Reference range	NSCP (n = 165) Mean ± SD	SCP (n = 71) Mean ± SD	t-statistic	*P* value (< 0.05)
Hb (g/dL)	11–16	14.1 ± 2.1	12.8 ± 2.2	4.198	0.000
RBC (x/L)	3.5–5.5	5.1 ± 0.7	4.7 ± 0.9	4.026	0.000
PCV (%)	37–54	40.8 ± 6.1	37.4 ± 6.3	3.854	0.000
MCV (fL)	80–100	79.2 ± 9.2	81.0 ± 8.4	− 1.387	0.167
MCH (pg)	27–34	27.8 ± 2.9	27.5 ± 3.3	0.652	0.515
MCHC (%)	32–36	34.6 ± 1.5	34.6 ± 6.3	0.078	0.938
WBC (x/L)	4–10	6.7 ± 2.7	9.5 ± 4.7	− 5.717	0.000
Neu (%)	50–70	59.5 ± 14.3	71.8 ± 16.3	− 5.779	0.000
MID (%)	3–14	8.1 ± 5.6	6.5 ± 3.2	2.338	0.020
Lym (%)	20–40	34.0 ± 20.1	20.5 ± 13.4	5.167	0.000
PLT (x /L)	100–300	234.9 ± 69.7	258.1 ± 96.1	− 2.085	0.038

The *P* value is provided for comparison between the NSCP and SCP groups

t-statistic shows the statistical difference between the means of NSCP and SCP

There were significant differences (P value < 0.05) between the hematologic abnormalities in the SCP and NSCP (Table 4). Secondly, in Table 4, the hematologic parameters for the two groups showed that there were more SCP with lower Hb, RBC, and PCV (anemia) than NSCP. A higher percentage of the SCP had increased WBC (leukocytosis) and neutrophils (neutrophilia) than the NSCP. In contrast, 43(61%) of SCP had lower lymphocyte counts (lymphocytopenia) than the NSCP. Bonferroni multiple-comparison correction test for Table 4 showed a p value of 0.007; all hematologic parameters with significant p value at < 0.05 were truly significant except for PCV.

**Table 4:** Categorization of hematologic abnormalities

	NSCP (n = 165)		SCP (n = 71)	
	n	%	n	%
*Hb (g/dL)*				
Anemia (< 11)	9	5.5	13	18.3
Normal (11–16)	126	76.4	56	78.9
Polycythemia (> 16)	30	18.2	2	2.8
*P* value (< 0.05)	< 0.001			
*RBC (x/L)*				
Anemia (< 3.5)	3	1.8	8	11.3
Normal (3.5–5.5)	124	75.2	55	77.5
Polycythemia (> 5.5)	38	23.0	8	11.3
*P* value (< 0.05)	< 0.001			
*PCV (%)*				
Anemia (< 37)	40	24.2	30	42.3
Normal (37–54)	123	74.5	41	57.7
Polycythemia (> 54)	2	1.2	0	0.0
*P* value (< 0.05)	0.016			
*WBC (x/L)*				
Leukopenia (< 4)	22	13.3	3	4.2
Normal (4–10)	122	73.9	41	57.7
Leukocytosis (> 10)	21	12.7	27	38.0
*P* value (< 0.05)	< 0.001			
*Neu (%)*				
Neutropenia (< 50)	45	27.3	9	12.7
Normal (50–70)	81	49.1	16	22.5
Neutrophilia (> 70)	39	23.6	46	64.8
*P* value (< 0.05)	< 0.001			
*Lym (%)*				
Lymphocytopenia (< 20)	26	15.8	43	60.6
Normal (20–40)	91	55.2	21	29.6
Lymphocytosis (> 40)	48	29.1	7	4.2
*P *value (< 0.05)	< 0.001			
*PLT (x/L)*				
Thrombocytopenia (< 100)	1	0.6	2	2.8
Normal (100–300)	142	86.1	54	76.1
Thrombocytosis (> 300)	22	13.3	15	21.1
*P* value (< 0.05)	0.109			

SCP (severe patients with COVID-19); NSCP (non-severe patients with COVID-19); Hb (hemoglobin); RBC (red blood cell); PCV (packed cell volume); MCV (mean corpuscular volume); MCH (mean corpuscular hemoglobin); MCHC (mean corpuscular hemoglobin concentration); WBC (white blood cell); Neu (neutrophils); MID (mid-range absolute); Lym (lymphocytes); PLT (platelet). The P value represents a comparison of the SCP and NSCP groups for each hematologic parameter, which consisted of patients with low, normal, and high values in each group. “Normal” refers to patients with values within the parameter reference ranges

### Diagnostic performance evaluation of target parameters

**Fig. 1:**
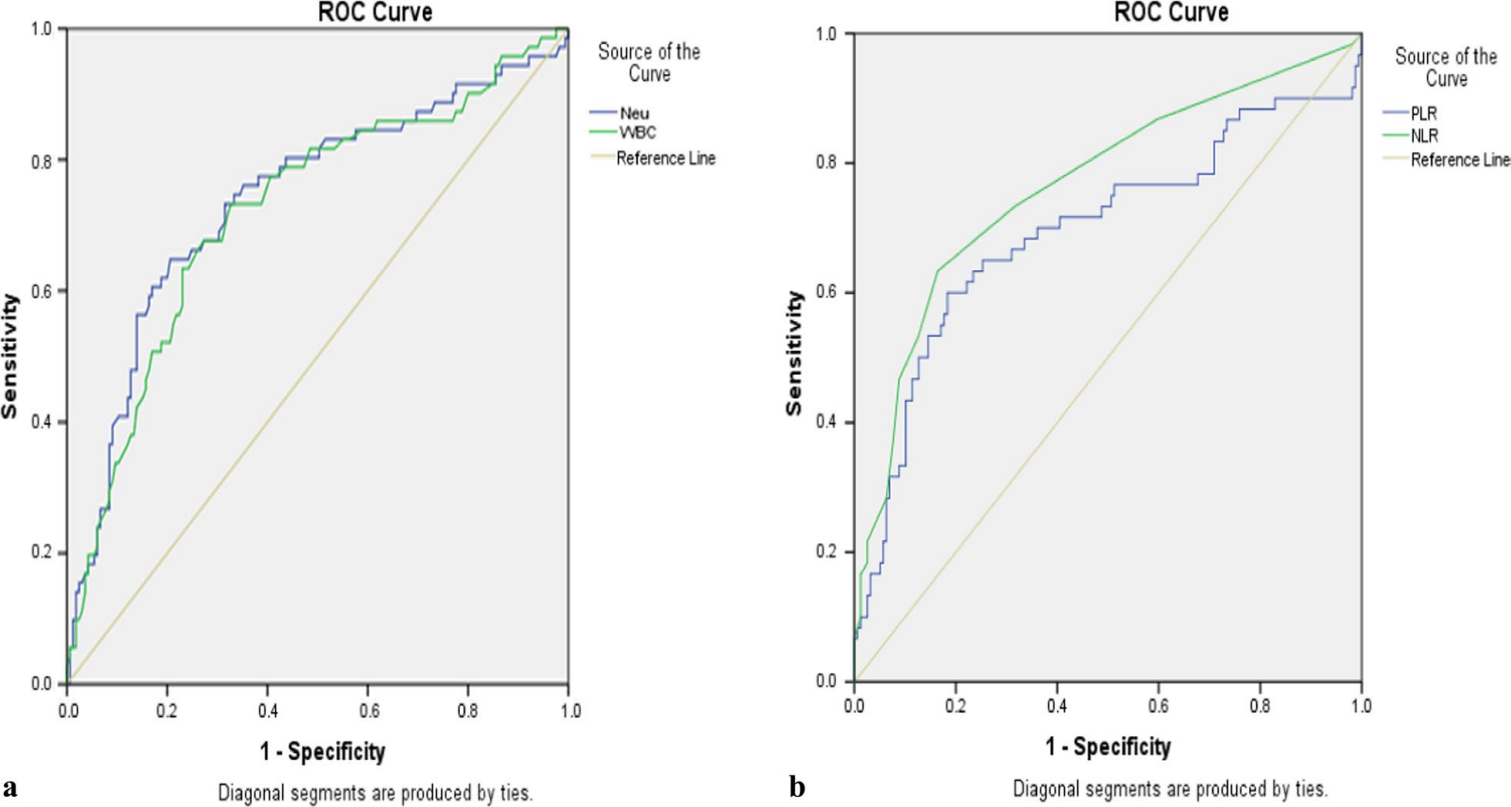
**a, b** Receiver operating characteristic (ROC) curve showing the relative diagnostic performances of neutrophils (Neu), white Blood cells (WBC), neutrophil–lymphocyte ratio (NLR), and platelet–lymphocyte ratio (PLR). The cutoff points detected for hematologic parameters with good performance were Neu 70.9, WBC 6.9, NLR 3.5, and PLR 166.1

The receiver operating characteristic (ROC) curve analysis was carried out to determine the significant differences in hematologic parameters between SCP and NSCP. The ROC curve plotted the values of sensitivity vs. 1-specificity as the value of the cutoff point moved from 0 to 1. It was observed that the ROC curves (the purple and green lines) were close to the top left corner of the plot, which indicated that the model was a good predictor as to whether the participants would have normal or abnormal neutrophil and WBC counts (Fig. 1).

The area under the curve (AUC) was an effective way to summarize the overall diagnostic accuracy of the test. Values within the range of 0–1 were designated as 0.7–0.8 acceptable; 0.8–0.9 excellent; and > 0.9 outstanding (Jayawant 2010). Among the parameters, neutrophils and WBC showed good diagnostic performances according to ROC analysis (Fig. 1a, Table 5). Neutrophils showed an AUC value of 0.739 with a cutoff point 70.9, sensitivity 62.0%, and specificity 80.0% (95% CI 0.665–0.813). The AUC value of 0.739 suggested a 73.9% chance that one could correctly distinguish a patient with normal neutrophil counts from a patient with abnormal neutrophil counts. Also, WBC showed an AUC value of 0.722 with a cutoff point 6.935, sensitivity 73.2%, and specificity 61.2% (95% CI 0.648–0.796). The AUC value of 0.722 indicated a 72.2% chance that one could correctly distinguish a patient with normal WBC counts from a patient with abnormal WBC counts (Table 5).

**Table 5:** Receiver operating characteristic analysis of promising markers for severity of COVID-19

Parameters	AUC	*P* value	OR (95% CI)		Cutoff	Sensitivity (%)	Specificity (%)
			Lower bound	Upper bound			
Neu	0.739	0.000	0.665	0.813	70.9	62.0	80.0
WBC	0.722	0.000	0.648	0.796	6.9	73.2	61.2
NLR	0.766	0.000	0.690	0.842	3.5	63.3	83.5
PLR	0.695	0.000	0.606	0.783	166.1	61.7	77.8

AUC, area under the curve; OR, odds ratio; Neu, neutrophils; WBC, white blood cells; NLR, neutrophil–lymphocyte ratio; PLR, platelet–lymphocyte ratio

The neutrophil–lymphocyte ratio showed an AUC value of 0.766 and a cutoff point of 3.5 with 63.3% sensitivity and 83.5% specificity (95% CI 0.690–0.842). The AUC value of 0.766 indicated a 76.6% chance of correctly distinguishing an NSCP from an SCP based on the neutrophil–lymphocyte ratio. The platelet–lymphocyte ratio showed a cutoff point of 166.1 with 61.7% sensitivity and 77.8% specificity (95% CI 0.606–0.783). The AUC value of 0.695 implied a 69.5% chance of correctly distinguishing an NSCP from an SCP based on platelet–lymphocyte ratio (Table 5). The odds ratio for Neu, WBC, NLR, and PLR that were < 1 indicated that these parameters were significant in patients with increased COVID-19 severity.

## Discussion

This was an observational study set to determine the significance of hematologic parameters in COVID-19 severity. The result of our study showed that patients with severe illness had abnormalities in their hematologic data, with significantly reduced hemoglobin levels, lymphocyte and RBC counts and significant increases in WBC, neutrophil, and platelet counts. These findings indicated that hemoglobin levels, lymphocyte, WBC, RBC, neutrophil, and platelet counts were associated with COVID-19 severity. Other studies have reported them to be promising markers for predicting COVID-19 severity (Amgalan and Othman 2020; Fan et al. 2020; Mehta et al. 2020).

NLR and PLR have been reported to be good prognostic tools for COVID-19 severity as well as independent factors associated with COVID-19 progression (Yang et al. 2020a, b). This current study also indicated that patients with COVID-19, who had an NLR of > 3.50 and PLR of > 166.12, were more likely to experience COVID-19 disease progression and severe clinical outcomes. The study by Yang et al. (2020a, b) showed an area under the curve for NLR as 0.841 and PLR as 0.784, and our results of NLR of 0.766 and PLR of 0.695 concurred that NLR and PLR may potentially become good prognostic markers for determining COVID-19 severity.

COVID-19 disease progression from non-severe to severe had been reported to be linked to comorbidities such as age, gender, hypertension, diabetes, and chronic kidney disease (Sanyaolu et al. 2020; Honardoost et al. 2021). Our study revealed that hypertension and diabetes were common comorbidities associated with COVID-19 severity and mortality. In agreement with our study, Wu et al. (2020) and Ge et al. (2021) also reported that these comorbidities were associated with severity or mortality in individuals with COVID-19.

In this study, though the RBC and Hb levels of the SCP were found to be within the reference range, they were lower than those of the NSCP. This implied that the RBC and Hb levels tended toward the lower boundary of the reference range in patients with severe COVID-19. Our finding correlated with previous studies, which reported that patients with severe COVID-19 disease had significantly lower Hb levels (Guan et al. 2020; Huang et al. 2020; Young et al. 2020; Tao et al. 2021). A meta-analysis conducted by Hariyanto and Kurniawan (2020) revealed that some studies (Cai et al. 2020; Ji et al. 2020; Xu et al. 2020; Zhang et al. 2020a, b) defined anemia as Hb levels below 13 g/L. Although our study defined anemia as Hb levels of below 11 g/L, the mean value of Hb (12.83 ± 2.20) in the SCP aligned with the defined levels in these studies.

The frequency of leukocytosis, neutrophilia, and thrombocytosis was pronounced among the SCP when compared with the NSCP. This frequency shown by leukocytosis and neutrophilia was in tandem with multiple studies (Fan et al. 2020; Amgalan and Othman 2020). However, there was a contrast between our report on the increase in thrombocytosis with reports by Bao et al. (2020), Huang et al. (2020), and Yang et al. (2020a, b), which showed an association between thrombocytopenia and COVID-19 severity instead, citing the probability of thrombocytopenia being a marker for COVID-19 severity.

The NSCP showed significantly lower WBC and neutrophil counts than SCP, with a greater percentage of patients in the SCP presenting with neutrophilia. An increase in blood leukocytes is a marker for the presence of infections (Nilsson et al. 2014). Our study also revealed that members of the SCP group had higher leukocyte counts than the NSCP group, which correlated with a study done by Sun et al. (2021).

Studies had shown that although an increase in neutrophil counts is a marker for bacterial infection (Song et al. 2021; Rosales 2018), COVID-19 was also found to be responsible for high neutrophil counts, especially in those with severe outcomes (Reusch et al. 2021). These increases in neutrophil counts had been proposed to be due to cytokine storm and hyperinflammation (Zini et al. 2020; Rahman et al. 2021). It had been reported that COVID-19 had serious effects in the reduction in lymphocytes, probably due to cytokine-related apoptosis of the CD8 + T cells (Chen and Wherry 2020). Lymphocytopenia had been shown to be frequent among patients with severe COVID-19, with the majority of them having their lymphocyte counts decreasing with an increase in disease severity. Our study revealed significantly lower lymphocyte counts among the SCP compared to the NSCP, which was similar to a study by Zhang et al. (2020a, b) and Illg et al. (2021).

Peripheral blood neutrophil-to-lymphocyte ratio (NLR) is an indicator of the balance between systemic inflammation and adaptive immunity, which plays a vital role in the prediction of disease severity in several medical conditions (Faria et al. 2016). An increase in neutrophil–lymphocyte ratio signifies increased severity and possibly mortality in an individual (Song et al. 2021). An increase in neutrophil–lymphocyte ratio depicts an increase in neutrophil counts with a decrease in lymphocyte counts (Palladino 2021). This could be a result of the ability of increased neutrophils to elicit immunoregulatory granulocytic myeloid-derived suppressor cells from the bone marrow, which suppresses lymphocyte counts and operation (Aarts et al. 2019). A correlation between COVID-19 disease severity and increased NLR and PLR values had been reported (Palladino 2021). The receiver operating characteristics shown for neutrophils, WBC, NLR, and PLR indicated that such parameters had good predictive performance and could be a reliable diagnostic marker for COVID-19 severity.

Men and women had been reported to have significant disparities with respect to the prevalence and severity for a variety of viral infections. This may be explained in part by biological differences in antiviral, inflammatory, and cellular immune responses to viruses (Ueyama et al. 2020). Our study revealed that the SCP consisted of more men than women. This was in correlation with the fact that respiratory tract infectious diseases were more severe in men and, consequently, resulted in a greater fatality rate in men (Pijls et al. 2021). Hence, it is important to understand the epidemiology of gender differences in susceptibility and vulnerability to a certain infection outbreak; this would enable an effective response to or adequate preparation for the public health crisis by reducing the impact of the outbreak on health, economic, and social impact (Smith 2019).

Previous studies had reported an association between age and disease severity (Du et al. 2020; Cannistraci et al. 2021). In this current study, the mean age revealed that most patients in the SCP group were 50 years and above, while male patients above 60 years of age constituted the majority of patients with severe symptoms. This finding aligned with a study by Cannistraci et al. (2021) whose report from three European countries revealed that males who were 60 years and above showed significantly higher COVID-19 infection as well as death risk than females.

Malaria had been associated with COVID-19 due to the similarity in symptom presentations like severe anemia, head and body ache as well as fever; this had led to misdiagnosis, especially in malaria-endemic areas (WHO 2020). From our study, only about 3% of those who tested for malaria had positive results indicating little influence of malaria on hematology outcomes in this study. This finding was in agreement with the study by Amoo et al. 2020 who reported no association between COVID-19 and malaria in an urban setting. This low rate of malaria infection in our study could also be a result of the study location, being an urban setting with a low prevalence of malaria.

## Limitations

The limitations of this study included the fact that patients’ hematologic data after recovery were not sampled and our sample size was relatively small and was carried out in a single institution.

## Conclusions

COVID-19 infection affected the levels of hemoglobin, RBC, PCV, neutrophils, and lymphocytes, and the differences were significant between the SCP and NSCP groups. The significant changes in neutrophil and lymphocyte counts could assist in the prognosis and management of COVID-19 severity. Furthermore, NLR and PLR may be useful as prognostic tools for severe COVID-19 infection, as well as provide an objective basis for early identification and management in low-resource settings.

## Data Availability

All data generated or analyzed during this study are included in this published article. The raw data are available from the authors upon request.

## Abbreviations

SCP: Severe patients with COVID-19
NSCP: Non-severe patients with COVID-19
ROC: Receiver operating characteristics
WBC: White blood cells
PCV: Packed cell volume
RBC: Red blood cells
MCHC: Mean corpuscular hemoglobin concentration
AUC: Area under curve
NLR: Neutrophil–lymphocyte ratio
PLT: Platelets
MCV: Mean corpuscular volume
Hb: Hemoglobin
